# Evaluating Laser Haemorrhoidoplasty as a Short-Term Approach to Advanced Haemorrhoidal Disease

**DOI:** 10.7759/cureus.91008

**Published:** 2025-08-26

**Authors:** Chin Kiat Tan, Shou Kee Ng, Arijit Mukherjee

**Affiliations:** 1 General Surgery, University Hospital Hairmyres, Glasgow, GBR

**Keywords:** coloproctology, haemorrhoidal disease, laser haemorrhoidoplasty, medical technology adoption, therapeutic interventions

## Abstract

Background

Haemorrhoids are a benign anorectal condition that can significantly impact the quality of life of patients. Well-established treatments for advanced haemorrhoidal disease are not without complications. Laser haemorrhoidoplasty (LHP) is a less invasive non-excisional surgical technique that has fewer complications than conventional management.

Methodology

A retrospective study of all patients who received LHP for symptomatic Grade 3 and Grade 4 haemorrhoids from November 2017 to March 2022 in a district general hospital was conducted to assess short-term functional outcomes. Patients were asked to report the severity of pain on the Visual Analogue Scale and frequency of symptoms based on the Haemorrhoidal Severity Score (HSS developed by Nyström) two months before and after LHP. Participant consent and feedback were obtained either by a written questionnaire or a phone interview.

Results

A total of 57 patients were interviewed. The most common symptoms were bleeding (n = 54), prolapse needing reduction (n = 45), pain (n = 43), itching and discomfort (n = 28), and soiling (n = 24). There was a significant improvement in the severity of pain (p < 0.0001) postoperatively. Patients reported an improvement of their pre-existing symptoms, including pain (p < 0.00001), itching and discomfort (p = 0.0001), bleeding (p < 0.00001), soiling (p = 0.00007), and prolapse (p < 0.00001) based on HSS.

Conclusions

Evidence from this study suggests that LHP provides significant short-term improvement in the severity of symptoms for patients with symptomatic third- and fourth-degree haemorrhoids. While the results of this study are encouraging, larger prospective multicentre studies assessing long-term outcomes will be desirable for proper evaluation of LHP with respect to its efficacy and cost-effectiveness.

## Introduction

Haemorrhoids are a common anorectal disease with an estimated prevalence of 10%, as quoted by the National Institute for Health and Care Excellence, UK [1]. Patients with symptomatic haemorrhoids are frequently referred to secondary care due to their often-debilitating effects on quality of life when conservative measures fail. A thorough clinical assessment helps the surgeon determine whether operative management is indicated for symptomatic haemorrhoids when other non-invasive measures, such as rubber-band ligation, fail. This is typically based on Goligher’s classification for internal haemorrhoids [2]. Conventionally, all patients with Grade 3 and 4 haemorrhoids and those with Grade 2 haemorrhoids for which conservative measures fail qualify as such.

Understanding of the pathophysiology of haemorrhoidal disease has helped surgeons develop various operative methods to treat symptomatic haemorrhoids. The sliding anal cushion theory postulates that internal haemorrhoids arise when the supporting structures that keep the anal cushions in place lose elasticity [3]. Aggravating factors such as altered bowel habitus, anal hypertonia, and pregnancy increase intra-abdominal pressure, leading to reduction of venous return, congestion, and, hence, engorgement of the internal haemorrhoidal plexus [4]. This eventually causes bleeding due to mucosal trauma and prolapse, leading to tenesmus, soiling, itching, and discomfort. Pain is less common due to the absence of nerve fibres above the dentate line, but can arise following complications such as thrombosis and strangulation, and is quite commonly seen in patients with symptomatic external haemorrhoids.

Regardless, well-established options for advanced haemorrhoidal disease, such as open haemorrhoidectomy, stapled haemorrhoidopexy and transanal haemorrhoidal dearterialisation (THD), are not without complications. These include increased postoperative pain, symptomatic recurrence, anal stenosis, and sphincter dysfunction [5,6]. Postoperative pain was noted in 70-75% of patients who underwent open haemorrhoidectomy, 5-20% in stapled haemorrhoidopexy, and 2-20% in THD [7]. In contrast, laser haemorrhoidoplasty (LHP) is a relatively new non-excisional surgical technique that has been acclaimed in European studies to provide better short-term treatment outcomes in terms of symptom reduction and improved quality of life [8-11]. It operates on the principle of localised and controlled laser application submucosally into the haemorrhoidal mass to cause tissue shrinkage. It is understood that LHP has similar postoperative complications to the well-established options. A meta-analysis by Longchamp et al. [11] has shown that LHP has lower complication rates and is also learning curve dependent. The learning curve has been shown by Naderan et al. to be approximately three to five proctored cases [12]. The main aim of this study was to ascertain the effectiveness of LHP in reducing haemorrhoid symptoms, especially pain.

This article was previously presented as a meeting abstract at the 2020 AANS Annual Scientific Meeting on April 27, 2020.

## Materials and methods

A longitudinal cohort study was conducted retrospectively among all patients with refractory Grade 2, Grade 3, and Grade 4 haemorrhoids who received LHP in a district general hospital in the west of Scotland with a view to assessing short-term functional outcomes. Inclusion criteria were patients with refractory Grade II haemorrhoids or symptomatic Grade III and IV haemorrhoids not responding to conservative measures or recurring after open haemorrhoidectomy. Exclusion criteria were patients with inflammatory bowel disease, previous anal surgery (apart from procedures for haemorrhoids), and pregnant patients. These patients had LHP performed between November 2017, when the hospital first started offering it on the NHS, and March 2022 and were all included in this study. All patients were vetted preoperatively and had their procedures performed by the same surgical team consisting of two consultants and two senior registrars, all of whom had received formal training before performing LHP within the hospital. Afterwards, consent to participate in the study was obtained initially by an invitation letter. Feedback was obtained via a written questionnaire and later via a phone interview if replies were not received. Furthermore, all patients who underwent LHP were discharged with paracetamol and codeine postoperatively, and no local anaesthetic was used during the procedures. Patients were also discharged with a five-day course of oral metronidazole.

**Figure 1:**
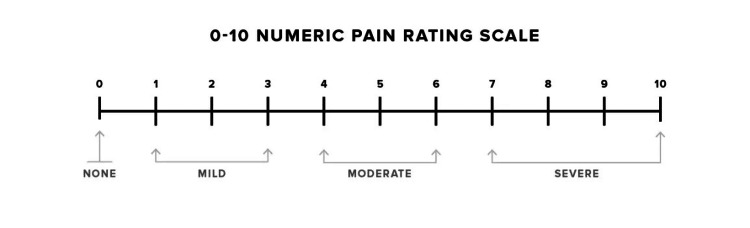
Numeric Rating Scale (NRS) for pain assessment. Mild pain is usually defined as a score between 1 and 3, moderate pain between 4 and 6, and severe pain between 7 and 10 [14].

Patients were asked to describe the frequency of their haemorrhoidal symptoms based on the Haemorrhoid Severity Score (HSS) developed by Nyström et al. [13], as shown in Table 1. Patients also rated the severity of pain based on the Numerical Rating Scale (NRS) [14] two months before and after LHP, as shown in Figure 1. The frequency of symptoms as per HSS was scored from 0, being the best functional outcome (i.e. no occurrence), to 3, being the worst functional outcome (i.e. daily occurrence). Pain severity was scored from 0, being no pain experienced, to 10, being the worst pain ever, as per NRS. The results pre- and post-LHP were collated and compared using statistical analysis. The study findings have been reported in line with the STROCSS criteria [15], and the study was registered with Research Registry (ID: researchregistry10788).

**Table 1:** Haemorrhoid Severity Score (HSS) as developed by Nyström et al. Points: never = 0; less than once a month = 1; less than once a week = 2; 1-6 times a week = 3; every day (always) = 4, as per Nyström et al. [13].

	0	1	2	3	4
How often do you have pain from the haemorrhoids?	Never	Less than once a month	Less than once a week	1–6 times a week	Every day (always)
How often do you have itching or discomfort in the anus?	Never	Less than once a month	Less than once a week	2–6 times a week	Every day (always)
How often do you have bleeding when passing a motion?	Never	Less than once a month	Less than once a week	3–6 times a week	Every day (always)
How often do you soil your underclothes (soiling from the anus)?	Never	Less than once a month	Less than once a week	4–6 times a week	Every day (always)
How often do you reduce a prolapsing haemorrhoid with your hand when passing a motion?	Never	Less than once a month	Less than once a week	5–6 times a week	Every day (always)

## Results

**Figure 2:**
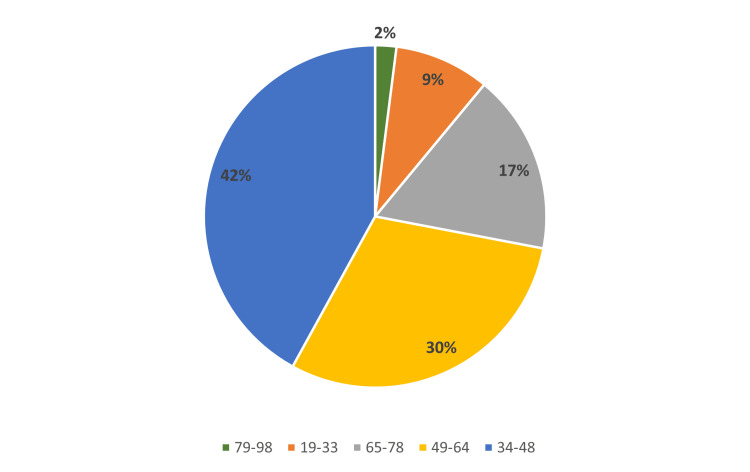
Age distribution of patients in this study.

A total of 57 patients were involved in this study, of whom 31 were male and 26 were female. The youngest patient was 24 years old, and the oldest was 85 years old. As shown in Figure 2, the majority of patients (n = 24) were in the 34-48-year age group, followed by the 49-64-year (n = 17) and 65-78-year (n = 10) age groups.

**Figure 3:**
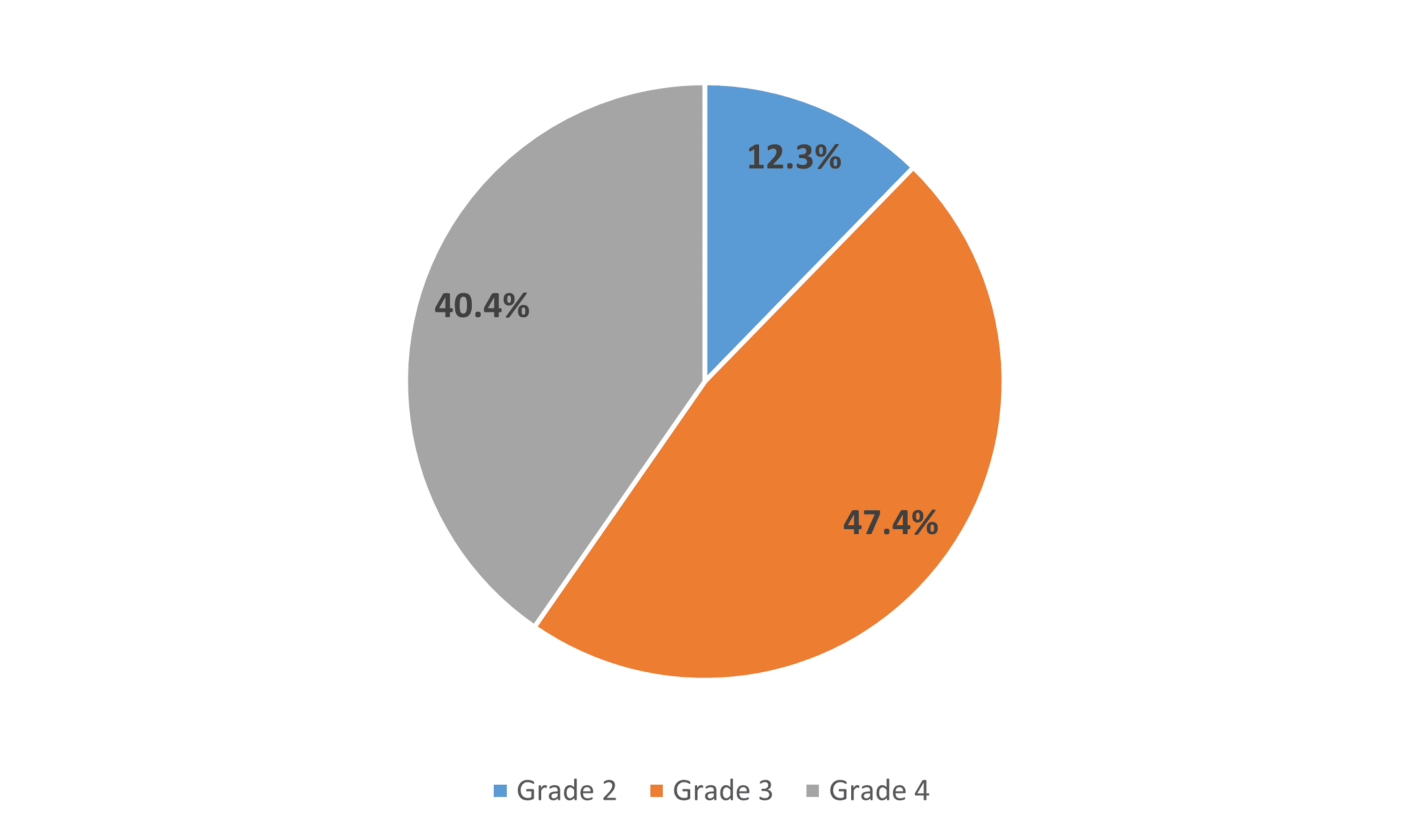
Haemorrhoidal grades of patients in this study.

Figure 3 shows the breakdown of the haemorrhoidal grades of patients within this study. Overall, 12.3% (n = 7) had Grade 1, 47.4% (n = 27) had Grade 2, and 40.4% (n = 23) had Grade 4 haemorrhoids.

**Figure 4:**
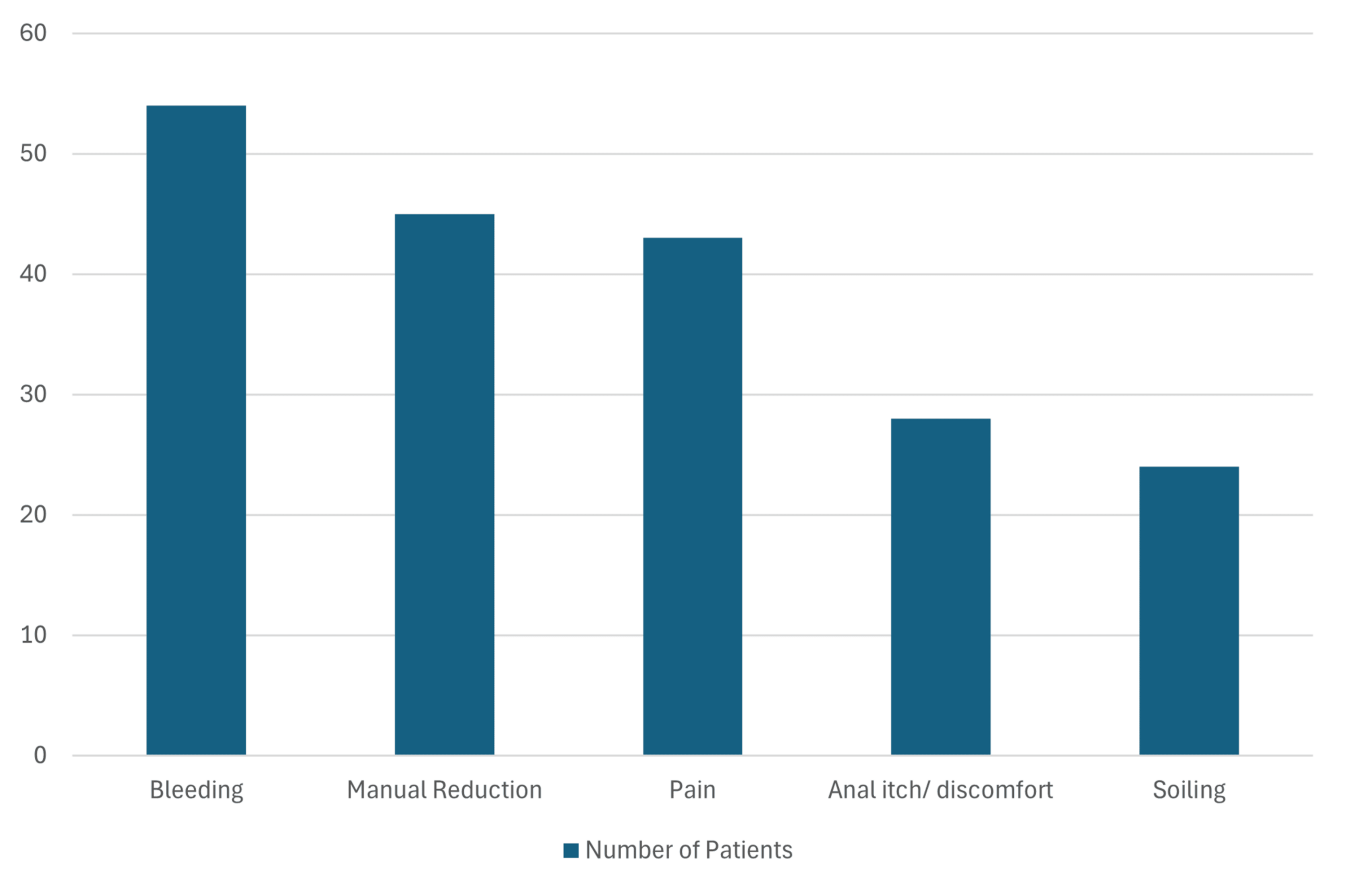
Frequency of haemorrhoid symptoms in our patient group.

Figure 4 shows that the most common symptom reported was bleeding (n = 54), followed by prolapse needing reduction (n = 45), pain (n = 43), itching and discomfort (n = 28), and soiling (n = 24). Within this cohort, previous banding treatment was utilised in 3 (42.9%) patients with Grade 2 haemorrhoids, 18 (66.7%) with Grade 3 haemorrhoids, and 15 (65.2%) with Grade 4 haemorrhoids.

**Figure 5:**
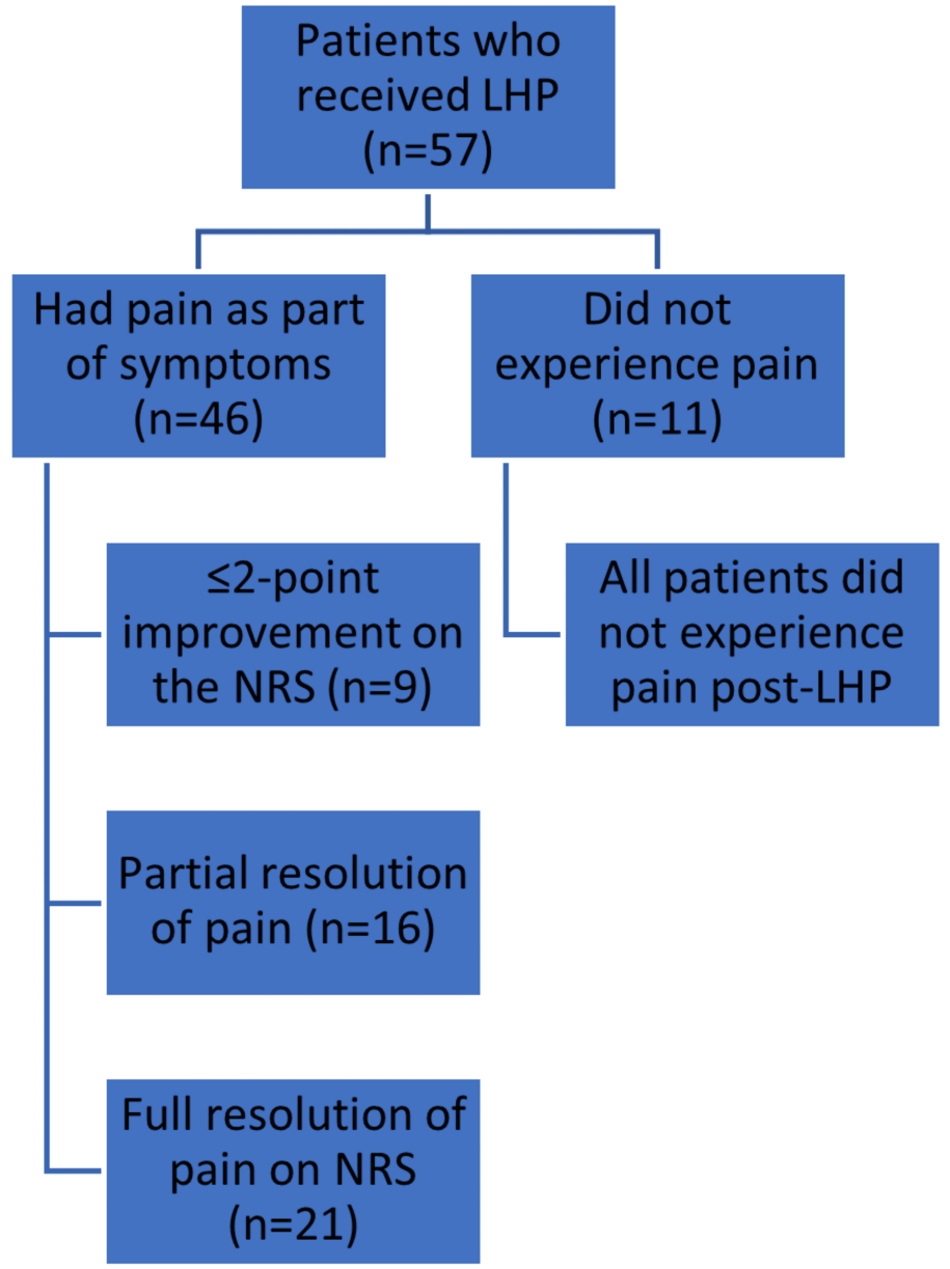
Flowchart depicting the number of patients still experiencing pain post-LHP. LHP = laser haemorrhoidoplasty; NRS = Numerical Rating Scale

As shown in Figure 5, 11 (19%) patients reported no pain with their symptomatic haemorrhoids two months before LHP. Most of the patients reported severe pain (n = 21, 36.8%), with 12 (21.1%) and 13 (22.8%) patients reporting moderate and mild pain, respectively, based on the NRS. Of the 46 patients who had pain as part of their symptoms, nine (19.6%) described a ≤2-point improvement on the NRS, while 21 (45.7%) reported full resolution (i.e. NRS = 0) of their pain two months after LHP.

**Figure 6:**
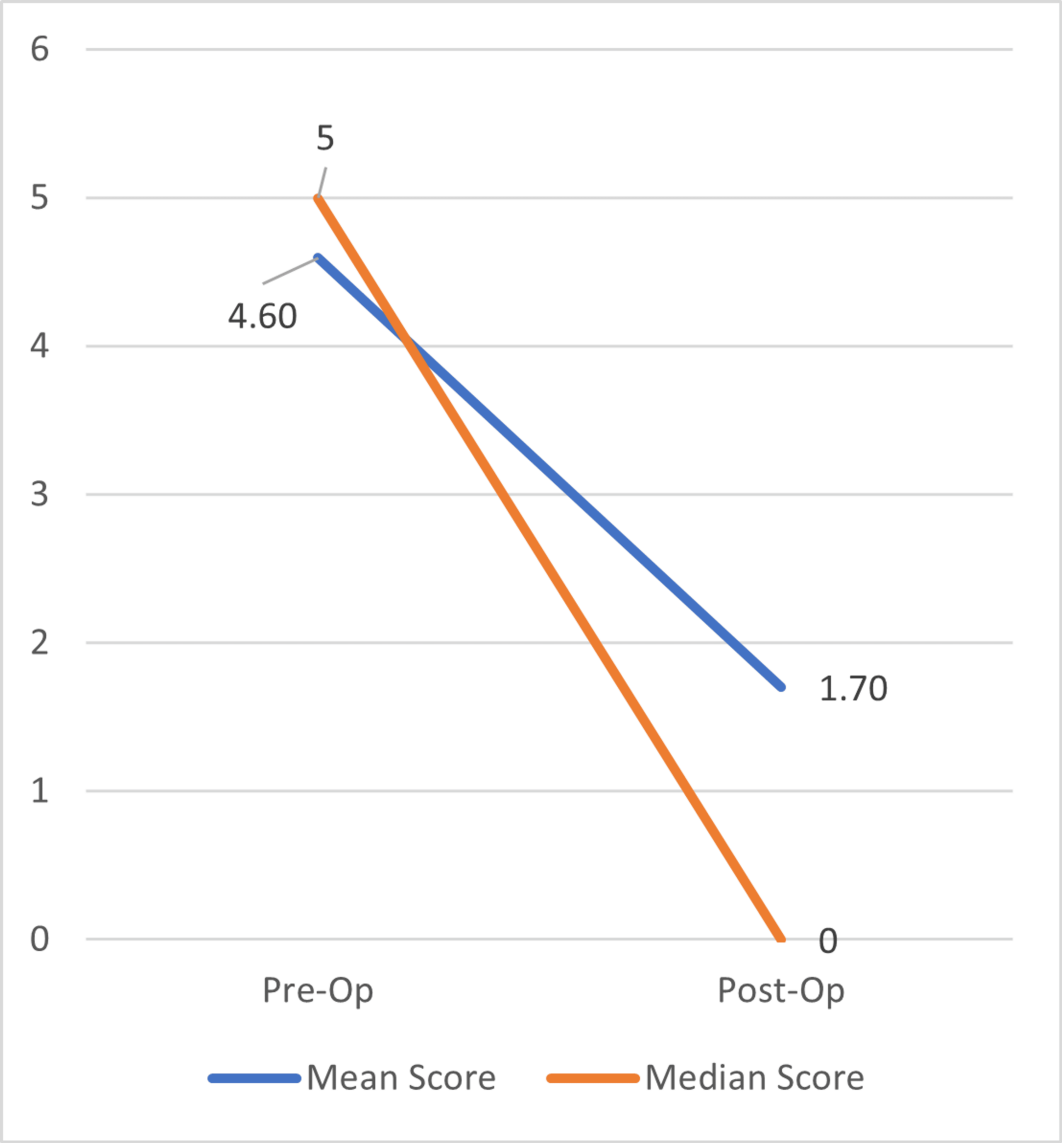
Improvement in mean and median pain scores post-LHP. LHP = laser haemorrhoidoplasty

Figure 6 shows that the mean score for pain based on the NRS dropped from 4.6 to 1.7, and the median score dropped from 5 to 0. Utilising paired t-tests for analysis, the significant improvement in the severity of haemorrhoidal pain two months post-LHP was statistically significant (p < 0.0001). Of note, the average decrease in postoperative pain after six weeks using the pain score for all three haemorrhoidal grades was fairly similar, with Grade 4 being the smallest drop at 2.7.

**Figure 7:**
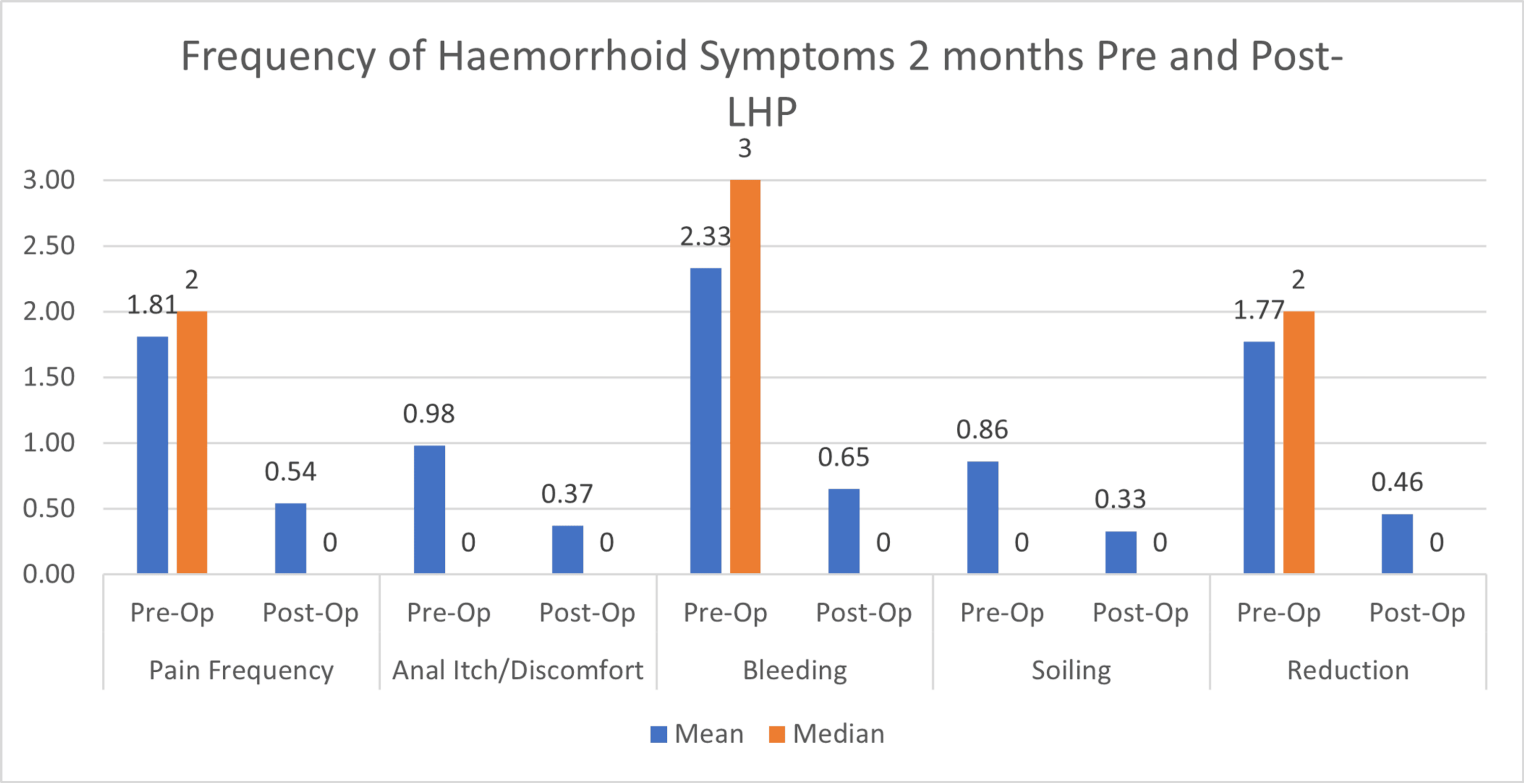
Improvement in mean and median scores for the severity of haemorrhoid symptoms, as defined by HSS. LHP = laser haemorrhoidoplasty; HSS = Haemorrhoid Severity Score

Overall, patients also reported significant improvement in the severity of their symptoms two months after LHP compared to how they felt two months before LHP, as shown in Figure 7. No intraoperative or immediate postoperative complications were observed in this cohort of patients. Following the paired t-test, there was statistically significant improvement in the severity of pain (p < 0.00001), anal itch and discomfort (p < 0.00001), bleeding (p = 0.0001), soiling (p = 0.00007), and prolapse needing manual reduction (p < 0.0001). These remained significant even after excluding patients who did not experience either of the five symptoms from each measure (all p < 0.0001).

## Discussion

An increasing number of local studies aim to study the effectiveness of LHP compared to other modalities, particularly focusing on postoperative outcomes, recurrence rates, and complications. The impression is that LHP involves shorter operating times with reduced postoperative pain and quicker resolution of normal activity [16], which strengthens the argument for LHP in favour of traditional haemorrhoidectomy. A single-centre study conducted in a similar manner to our study also published results showing significant improvements in the five classical haemorrhoidal symptoms, as described by HSS at three months [8,9]. Head-to-head trials comparing LHP and haemorrhoidectomy also showed that patients who had LHP reported faster resolution of pain and bleeding in the following weeks after surgery [8-11,16-18], which is consistent with our findings.

Despite the findings observed, there are some limitations to this study. First, this was a fairly small number of patients over a relatively extended period of time, largely due to the cancellation of all elective surgical activity in the United Kingdom during the COVID-19 pandemic. Additionally, this was a local study that was not subject to randomisation and control group comparison, and data were collected and analysed retrospectively. This study design increases the risk of selection bias, as in addition to non-randomisation of patients, some of the external studies referenced are also single-centre and observational, further contributing to this potential bias. Additionally, unmeasured confounding variables such as patient comorbidities and variations in postoperative care could have influenced the outcomes observed, limiting causal interpretation. Therefore, the outcomes observed may not be fully generalisable across broader patient populations or settings. To accurately assess LHP’s effectiveness, randomised controlled trials involving multiple centres will help reduce bias and validate the effectiveness of LHP as a treatment option. It will also be beneficial if prospective head-to-head studies comparing LHP with other surgical treatment options are conducted.

Second, this study has not formally explored the complication rates and recurrence rates of LHP, and hence, will not be able to decisively conclude how effective LHP is for treating symptomatic haemorrhoids compared to other established surgical options such as haemorrhoidectomy and transanal haemorrhoidal dearterialisation in the longer term. Furthermore, this will also affect how the results in this study will be generalisable across different settings. Recent studies have suggested the possibilities of LHP having higher [19,20] or equivocal [21] recurrence rates compared to open haemorrhoidectomy. It is also undeniable that LHD costs in both acquiring the equipment and maintenance are much higher than the conventional treatment methods. However, this should be weighed against the costs saved from a shorter postoperative inpatient stay, lower complication rate, and shorter operative time. Prospective larger multicentre studies assessing long-term outcome and complication rates will be desirable for proper evaluation of LHP with respect to its efficacy and cost-effectiveness.

## Conclusions

LHP appears to be a promising treatment modality for patients with symptomatic high-grade haemorrhoids, given its minimally invasive nature. Furthermore, LHP can be repeated if indicated and has been shown to have improved postoperative and short-term functional outcomes compared to open haemorrhoidectomy. However, it remains to be seen if it will supersede open haemorrhoidectomy as the operative option of choice to definitively treat symptomatic haemorrhoids. The results in this study, although favourable, must be interpreted with caution due to its short-term data. There remains a gap in studies directly comparing clinical outcomes of transanal haemorrhoidal dearterialisation with LHP, and it will be extremely beneficial to see how LHP performs in comparison with other minimally invasive treatment options.
